# A ^1^H-NMR approach to myocardial energetics

**DOI:** 10.1038/s41598-020-74241-3

**Published:** 2020-10-14

**Authors:** Jackie A. Heitzman, Tyler C. Dobratz, Kaleb D. Fischer, DeWayne Townsend

**Affiliations:** Department of Integrative Biology and Physiology, Medical School, University of Minnesota, 2231 6th St. SE, Minneapolis, MN 55455 USA

**Keywords:** Metabolomics, Heart failure

## Abstract

Understanding the energetic state of the heart is essential for unraveling the central tenets of cardiac physiology. The heart uses a tremendous amount of energy and reductions in that energy supply can have lethal consequences. While ischemic events clearly result in significant metabolic perturbations, heart failure with both preserved and reduced ejection fraction display reductions in energetic status. To date, most cardiac energetics have been performed using ^31^P-NMR, which requires dedicated access to a specialized NMR spectrometer. This has limited the availability of this method to a handful of centers around the world. Here we present a method of assessing myocardial energetics in the isolated mouse heart using ^1^H-NMR spectrometers that are widely available in NMR core facilities. In addition, this methodology provides information on many other important metabolites within the heart, including unique metabolic differences between the hypoxic and ischemic hearts. Furthermore, we demonstrate the correlation between myocardial energetics and measures of contractile function in the mouse heart. These methods will allow a broader examination of myocardial energetics providing a valuable tool to aid in the understanding of the nature of these energetic deficits and to develop therapies directed at improving myocardial energetics in failing hearts.

## Introduction

The continuous beating of the heart is essential for survival, to maintain this level of contractile function the heart maintains an extraordinary rate of metabolic activity, turning over its entire ATP pool every 2–10 s^1^. Remarkably, the healthy heart is able to maintain intracellular levels of key metabolites essentially constant across large ranges of metabolic activity^2,3^. The loss of this metabolic control is a hallmark of failing hearts with both reduced^4–8^ and preserved contractile function^9,10^. Heart failure is a growing epidemic affecting 26 million people across the world and has a 5 year mortality rate of greater than 40%^11,12^. Currently the only effective therapies are surgical replacement with a donor heart, providing a median survival of 10.6 years^13,14^. Developing a better understanding of the underlying causes of metabolic dysfunction in the failing heart may provide new therapeutic targets that support the energetics of the heart, resulting in improved systolic and diastolic function.

Within the failing heart there are significant changes in the substrate utilization, including a shift from the oxidation of lipids toward greater utilization of carbohydrates^15–17^. In addition to alterations of substrate preferences, there is a disruption of the creatine kinase system resulting in limitations in overall ATP consumption and production^6,18^ and likely a series of other metabolic differences. These studies highlight the importance of measuring the relative concentrations of multiple metabolites to fully understand the pathophysiology of the diseased heart. Changes in several metabolic pathways can have significant impact on ATP production and the poor energetics of the failing heart and a better understand how these pathways contribute to the disease process could greatly enhance the development of effective therapeutic approaches.

In the past few years, the field of metabolomics has exploded, with the development of new approaches and methods that allow for the simultaneous measures of dozens to hundreds of unique molecular entities from biological samples. Most of these approaches rely on mass spectrometry as the foundational technology. Mass spectrometry is excellent for the identification and relative levels of individual molecules in biological samples. However, it is difficult to compare the molar concentrations between molecules within a given sample; to do so requires calibration of each metabolite with isotopically labeled molecules which are expensive and often not readily available for study. In contrast, ^1^H-NMR spectroscopy provides a highly quantifiable method, allowing the comparisons of molecular concentrations within a given sample. This capability is particularly important in the evaluation of energetics within the heart, where the interplay of half a dozen molecules is necessary to provide a full snapshot of the energetic status at a given moment.

Here we present a method for the preparation and analysis of metabolites from the mouse heart. Using a ^1^H-NMR approach, we provide details for the measurement of over 20 individual molecules extracted from the mouse myocardium. This methodology is validated in both hypoxic and ischemic hearts model and calculated measures of cellular energetics are demonstrated to correlate with contractile function. This tool will be valuable for providing a detailed energetic picture of the contracting mouse heart.

## Materials and methods

### Animals

The mice used in this study were C57BL/10-SnJ male mice taken from colonies maintained at the University of Minnesota, derived from mice purchased from Jackson Laboratory. All animals used were male mice, 5.2 ± 0.1 months of age. All experiments were performed using methods defined by protocols that were reviewed and approved by the University of Minnesota Institutional Animal Care and Use Committee.

### Langendorff

Langendorff protocols were performed as described elsewhere^19^. Briefly, mice were euthanized with 100U of Heparin and 250 mg/kg of sodium pentobarbital. Hearts were excised and perfused on the Langendorff with modified Krebs buffer (in mM: 118 NaCl, 0.5 EDTA, 4.7 KCl, 1.2 MgSO_4_, 1.2 Na_2_HPO_4_, 15 glucose, 2.5 CaCl_2_, 25 NaHCO_3_, and 0.5 pyruvate). Oxygen was monitored throughout the protocol using flow through oxygen probes (Microelectrodes, Inc.) sampling both the perfusate and cardiac effluent. Coronary flow was monitored via an ultrasonic flow probe (Transonic) and left ventricular pressure was measured using a water filled balloon coupled to a solid-state pressure transducer (Millar Instruments). After a period of equilibration, the hearts either received an additional minute of continuous flow, 1 min of no-flow ischemia, or 10 min of hypoxia. During the hypoxia period cannula oxygen levels were 175 ± 11 mmHg compared to 524 ± 18 mmHg at baseline. At the end of the treatment period, hearts were quickly removed and snap frozen in liquid nitrogen cooled 2-methyl butane. Tissues were stored at − 80 °C until further analysis.

### Metabolite extraction

Polar metabolites were extracted using methods described previously^20^. Briefly, frozen tissues were pulverized, weighed, and homogenized in a mixture of methanol (4 ml/g_Wet Weight_) and water (0.85 ml/g_Wet Weight_); homogenization in methanol effectively halts all enzymatic activity. Care was taken to prevent any thawing of the sample prior to homogenization and all subsequent extraction steps were carried out a 4 °C. Once the tissue was homogenized, 150 nmol of 4,4-dimethyl-4-silapentane-1-sulfonic acid (DSS) was added to the solution. To the methanol homogenate, 2 ml/g_Wet Weight_ chloroform was added with vortexing. After through mixing another 2 ml/g_Wet Weight_ of chloroform and 2 ml/g_Wet Weight_ of water were added followed by vigorous mixing of each sample. Following a 15-min incubation on ice, the samples were then spun at 1000 RCF for 15 min at 4 °C resulting in a phase separation. The upper water/methanol layer was then transferred to a fresh tube and dried under a stream of nitrogen gas. Once dry the samples were stored at − 80 °C under nitrogen until resuspension for NMR spectroscopy.

### NMR

#### Standard preparation

ATP, ADP, AMP, creatine, and creatine phosphate were all purchased from Millepore-Sigma. For experiments to determine the ideal conditions for analysis, a standard mix of all five of these compounds were made up into an equimolar (0.5 mM) solution in 250 mM phosphate buffer (0.2% sodium azide and 20% D_2_O). NMR spectra were collected on a 700-MHz Bruker Avance NMR spectrometer with a 5-mm TXI cryoprobe. Spectra were acquired with a gradient-enhanced 1D NOESY pulse sequence (noesygppr1d) with presaturation of the water signal and the following acquisition parameters: 256–1024 scans, 8 dummy scans, 1.5 s recycle delay, 3.9 s acquisition time and 12 ppm spectral width. ^1^H 90° pulse width and transmitter offset were optimized for each sample. Spectra were processed with 0.3 Hz line broadening and zero-filled to 128 k data points.

#### Metabolomic samples

Dried metabolite samples were resuspended in a 250 mM phosphate buffer (0.2% sodium azide and 20% D_2_O). Unless stated otherwise, the pH of the buffer was adjusted to 8.1, to maximize the chemical shift difference between AMP and ADP/ATP peaks. 600 µl of each sample was transferred to 5 mm tubes and placed into the rack of a SampleJet autosampler coupled to the Bruker Avance 700 MHz NMR spectrometer. Spectra were collected using the protocol described above. Spectral signal to noise ratio was assessed by the presence of a clear NADP(H) peak at 9.29 ppm. If this peak was unclear additional scans were added. Once a proper signal to noise ratio was achieved, 20 µl of 10 mM EDTA in 250 mM phosphate buffer was added to each tube to fully separate the ADP and ATP peaks at ≈ 8.52 ppm.

#### NMR spectral analysis and peak assignment

Spectra were analyzed using the Chenomx NMR Suite (Chenomx, Edmonton, Alberta). Raw spectra were phase corrected using the automated algorithm, the removal of the residual water peak and the baseline correction were performed manually. Using the compound spectral library, preliminary analyses determined a list of compounds that were reliably detectable in extracted samples. Identified compounds needed to have at least one peak that had a distinct chemical shift that was separate from other signals. From this analysis a list of 24 compounds was identified and quantified in at least 4 samples in any of the treatment groups. Quantification used the internal DSS as a standard, peak areas were measured and corrected for the number of protons responsible for that signal by the Chenomx NMR suite. Heart weights were calculated based on the percentage of water present in hearts after 20 min of perfusion with or without 1 min of no-flow ischemia. Hearts with continued perfusion were 86.3 ± 0.5% water, hearts subjected to 1 min of no-flow ischemia had 80.8 ± 0.7% water. Using these corrections, dry heart weights were calculated and found not to differ significantly between treatment groups; 34 ± 2 mg for perfused hearts, 38 ± 2 mg for ischemic hearts, and 33 ± 1 mg for hypoxic hearts. Intracellular concentrations were determined using the assumptions that: (1) all of the metabolites detected arise from within the cytosol of the cardiac myocytes and (2) the cytoplasmic volume of the perfused heart is 2.43 times the dry weight of the heart^21^, this latter value was obtained using NMR based tracer studies of perfused hearts.

### Metabolomic analysis

Concentrations determined from the Chenomx analysis were analyzed using a custom R script^22^. This script calculated the principle component analysis using the base prcomp command and heatmap using the heatmap3 library. All plots were generated using ggplot2^23^.

### Cellular energetic calculations

The adenosine nucleotide energy charge, first described by Atkinson and Walton^24^, provides an estimate of cellular energy using the concentrations of ADP, AMP, and ATP.

$$Energy\;Charge= \frac{\left[ATP\right]+0.5[ADP]}{\left[ATP\right]+\left[ADP\right]+[AMP]}$$

However, in muscle tissue direct measurement of ADP and AMP is confounded by the fact that significant portions of these metabolites are bound to proteins. These bound forms do not contribute to the thermodynamic equilibrium of ATP hydrolysis. Free ADP concentrations can be calculated by assuming that the creatine kinase reaction is at equilibrium^25,26^:

$${[ADP]}_{Free}=\frac{[ATP][Cr]}{[PCr][{H}^{+}]{K}_{CK}}$$

Using K_CK_ as 1.66 × 10^9^ the thermodynamically relevant concentration of ADP can be determined^27^. Similarly, free AMP can be calculated using the adenylate kinase equilibrium reaction, with a K_AK_ of 0.952^26^ and the following formula:

$${[AMP]}_{Free}=\frac{{K}_{AK}{{[ADP]}_{Free}^{2}}}{[ATP]}$$

The energy released with the hydrolysis of ATP is the currency the myocardium uses to perform its systolic and diastolic functions. The free energy of ATP hydrolysis, ∆G_ATP_ is determined using this formula:

$${\Delta G}_{ATP}={\Delta G}_{obs}^{\circ}+RT ln\frac{{[ADP]}_{Free}[{P}_{i}]}{[ATP]}$$

The baseline inorganic phosphate in Langendorff perfused hearts is 5.0 ± 0.3 mM and increases to 17.5 ± 1.5 mM after 2 min of ischemia^21^ or to 7.5 ± 1.1 mM with 10 min of hypoxia^28^. $\Delta {G}_{obs}^{\circ }$ is − 30.5 kJ mol^−1^. The gas constant, R, is 8.31 J/K mol^−1^, and the absolute temperature was 310 K.

## Results

**Figure 1:**
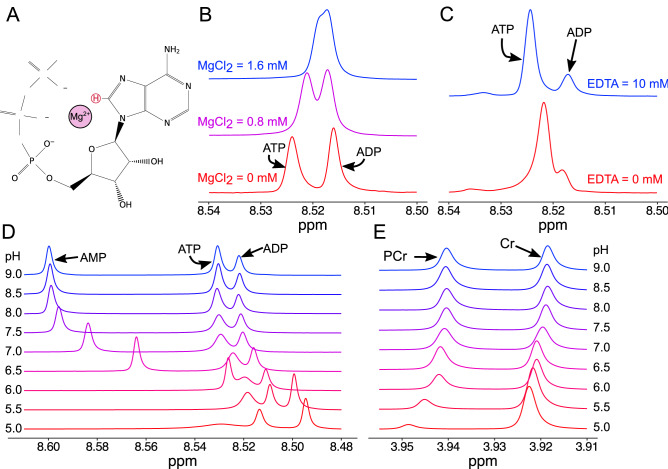
Defining the conditions to allow the quantification of purine nucleotides from extracts of cardiac muscle. (**A**) Schematic of ATP with Mg^2+^ ion. The proton used for distinct quantification is highlighted in red. (**B**) The effect of Mg^2+^ ion concentration in equimolar concentrations of ATP and ADP. The presence of Mg^2+^ alters the chemical shift of the ATP proton, but not the ADP; resulting in a convergence of these peaks with physiological concentrations of Mg^2+^. (**C**) ^1^H-NMR spectra from polar metabolites extracted from perfused mouse hearts without (red) and with EDTA (blue). In the presence of EDTA, the ATP and ADP peaks are clearly distinct and easily quantifiable. (**D**) The effect of extract pH on the separation of the purine proton peaks, note the high mobility of the AMP proton. (**E**) The pH sensitivity creatine (Cr) and phosphocreatine (PCr) protons.

An important goal of this approach is to provide a detailed assessment of molecules important in defining the energetic status of the myocardium. The adenosine nucleotides (ATP, ADP, and AMP) are central to this capability. In preliminary studies, it was found that the ^1^H-NMR peaks of the adenosine nucleotides were often highly overlapping, but that this overlap varied between tissues. The ability of ATP to chelate the divalent cation Mg^2+^ is well known, we hypothesized that this chelation would result in a significant shifting of the electronic environment near the proton responsible for the ^1^H-NMR signal used to separate the adenosine nucleotides (Fig. 1A). To test this possibility, we collected ^1^H-NMR spectra of equimolar amounts of ADP and ATP in the presence and absence of physiological levels of Mg^2+^. These studies demonstrated that the separation of the ADP and ATP peaks is eliminated in a dose responsive manner in the presence of MgCl_2_ (Fig. 1B). The importance of the presence of Mg^2+^ in physiological samples was confirmed by the inclusion of the divalent cation chelator EDTA. In the absence of EDTA, there was often a significant degree of overlap between the peaks of ADP and ATP. Addition of EDTA, resulted in a marked increase in the separation of these two peaks, allowing for the identification and quantification of these important molecules (Fig. 1C).

In addition to variability in the peak separation of ADP and ATP, it was noted in preliminary studies that the chemical shift of the AMP proton was highly variable. Prior studies demonstrated that the AMP proton shift was not affected by the presence of Mg^2+^, however, examination of the AMP structure suggested that the charge status of the alpha-phosphate group may modulate the electronic environment around the AMP proton. It was hypothesized that changes in the pH may modify the chemical shift of the AMP proton. To test this hypothesis, ^1^H-NMR spectra were collected in buffers with equimolar amounts of AMP, ADP, and ATP with different pH conditions. These studies revealed a pH-dependent mobility of the chemical shift of the AMP proton, such that at more acidic pH the AMP peak overlapped the ADP and ATP peaks (Fig. 1D). Additional studies also examined the pH dependence of the phosphocreatine (PCr) and creatine (Cr) peaks. These results demonstrated that the chemical shift and peak amplitude are highly dependent on the pH of the buffer and that hydrolysis of PCr to Cr readily occurs at acidic pH levels (Fig. 1E). Maintaining samples at neutral to slightly alkaline pH maximizes the stability of PCr and maximizes the peak separation of the AMP proton with the peaks of ADP and AMP.

**Figure 2:**
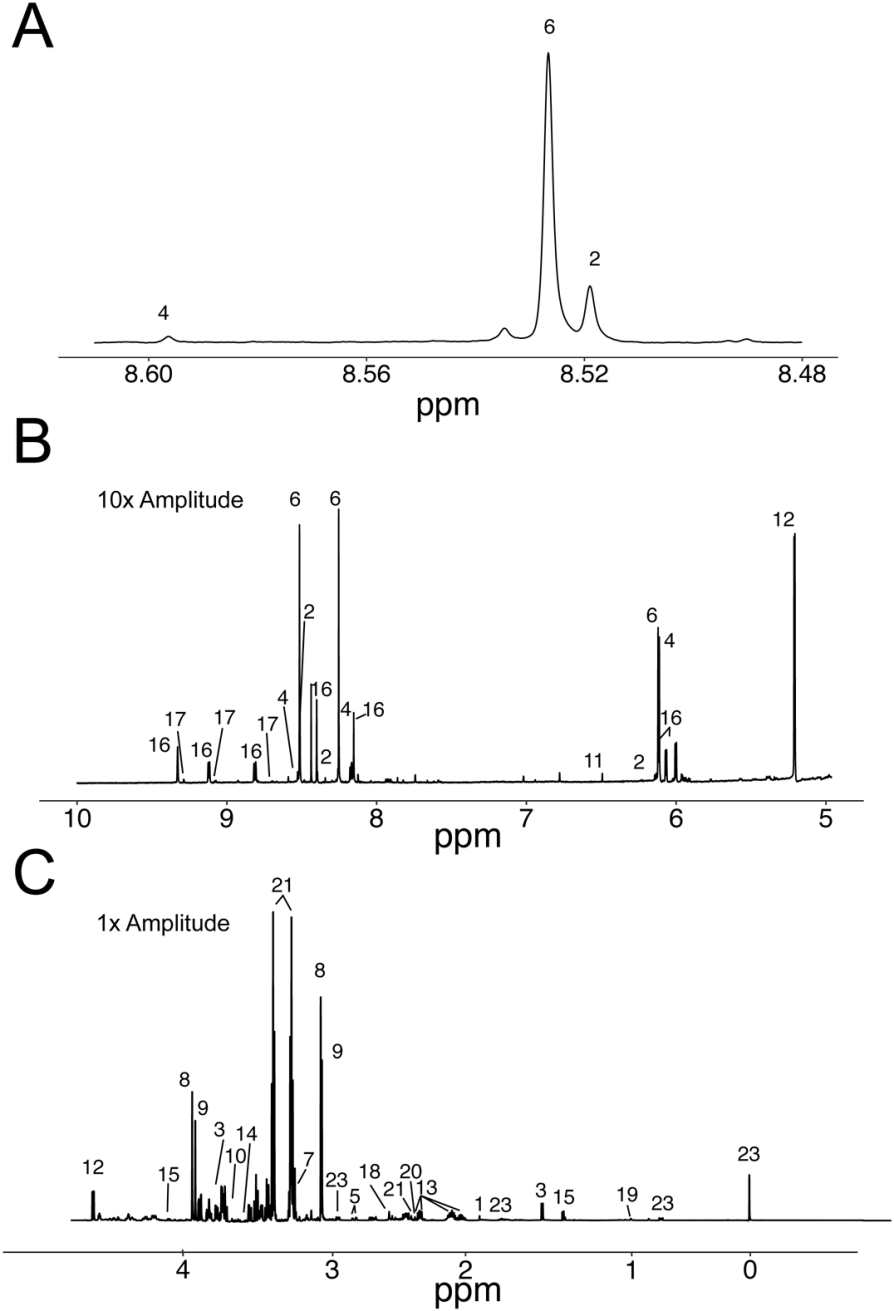
Representative ^1^H-NMR spectrum of extracted polar myocardial metabolites. Peaks are labeled as: 1. Acetate; 2. ADP; 3. Alanine; 4. AMP; 5. Aspartate; 6. ATP; 7. Carnitine; 8. Phosphocreatine; 9. Creatine; 10. Ethylene Glycol; 11. Fumarate; 12. Glucose; 13. Glutamate; 14. Glycine; 15. Lactate; 16. NAD(H); 17. NADP(H); 18. Oxalacetate; 19. Pantothenate; 20. Pyruvate; 21; Succinate; 22. Taurine; 23. DSS. Note the spectra containing the residual water peak (4.70 to 4.85 ppm) is not shown. (**A**) Demonstrates the spectral region of adenine proton following addition of EDTA, note separation between ATP (6) and ADP (2). (**B**) Spectra represent peaks above the water peak (> 4.8 ppm) are multiplied by 10 to allow peak detail to be observed. (**C**) Represent peaks below 4.70 ppm.

Using the protocol described here, we reliably measured the concentration of 24 metabolites in isolated perfused hearts standardized to a known concentration of sodium trimethylsilylpropanesulfonate (DSS). The peaks used for quantification are shown in Fig. 2. Quantified data are presented in Table 1. Not surprisingly, many of these metabolites are significantly altered by 1 min of no flow ischemia or 10 min of hypoxia. Many of these changes are expected because of marked reduction in oxygen availability, such as reductions in phosphocreatine and ATP and increases in lactate and succinate. Importantly, the concentrations of many of the most labile metabolites such as ATP and phosphocreatine are similar to other studies using ^31^P-NMR and biochemical approaches^28–31^.

**Table 1:** Metabolite concentrations from control and ischemic hearts.

Metabolite	Control	Ischemia	Hypoxia	Stat
Acetate	0.48 ± 0.04 (9)	0.35 ± 0.02 (8)	0.50 ± 0.02 (7)	a,c
ADP	1.83 ± 0.19 (9)	2.59 ± 0.33 (8)	3.13 ± 0.17 (7)	b
Alanine	2.74 ± 0.32 (9)	3.00 ± 0.40 (8)	4.87 ± 0.50 (7)	b,c
AMP	0.23 ± 0.05 (9)	0.94 ± 0.18 (8)	2.04 ± 0.24 (7)	a,b,c
Aspartate	2.80 ± 0.27 (9)	1.71 ± 0.20 (8)	2.68 ± 0.23 (6)	a,c
ATP	9.00 ± 0.69 (9)	5.29 ± 0.44 (8)	4.90 ± 0.41 (7)	a,b
Carnitine	1.95 ± 0.19 (7)	1.15 ± 0.13 (8)	1.10 ± 0.29 (4)	a,b
Choline	0.09 ± 0.02 (4)	0.07 ± NA (1)	0.12 ± 0.05 (3)	
Creatine	15.54 ± 1.30 (9)	19.25 ± 2.26 (8)	24.67 ± 1.21 (7)	b
Ethylene glycol	0.13 ± 0.03 (9)	0.22 ± 0.03 (8)	0.18 ± 0.01 (6)	
Fumarate	0.16 ± 0.05 (9)	0.34 ± 0.05 (8)	0.70 ± 0.05 (7)	a,b,c
Glucose^1^	7.40 ± 0.60 (9)	3.10 ± 0.30 (8)	9.01 ± 0.37 (7)	a,c
Glutamate	8.24 ± 0.72 (9)	6.42 ± 0.68 (8)	5.55 ± 0.32 (7)	b
Glycine	0.96 ± 0.27 (9)	0.41 ± 0.12 (8)	2.30 ± 0.56 (7)	b,c
IMP	ND	0.03 ± 0.00 (2)	0.23 ± 0.04 (5)	–
Lactate	1.67 ± 0.31 (9)	10.24 ± 1.66 (8)	4.44 ± 0.40 (7)	a,c
NAD(H)	2.44 ± 0.22 (9)	1.84 ± 0.19 (8)	2.10 ± 0.10 (7)	
NADP(H)	0.19 ± 0.02 (9)	0.14 ± 0.02 (8)	0.17 ± 0.02 (7)	
Oxaloacetate	2.14 ± 0.29 (9)	1.46 ± 0.20 (8)	2.54 ± 0.28 (6)	c
Pantothenate	0.05 ± 0.01 (7)	0.04 ± 0.01 (4)	0.07 ± 0.01 (2)	
Phosphocreatine	18.32 ± 2.02 (9)	5.21 ± 0.62 (8)	5.63 ± 0.37 (7)	a,b
Pyruvate^1^	0.11 ± 0.01 (9)	0.03 ± 0.00 (8)	0.11 ± 0.01 (7)	a,c
Succinate	0.25 ± 0.04 (9)	0.84 ± 0.17 (8)	1.32 ± 0.25 (7)	a,b
Taurine	85.23 ± 5.89 (9)	71.34 ± 6.20 (8)	88.61 ± 5.32 (7)	

Data are presented an mM assuming cytosolic distribution.

^1^Glucose and pyruvate are present in the perfusate, their concentration assumes uniform extracellular and cytosolic distribution.

Data mean ± SEM (n); a: P < 0.05 between Control and Ischemia; b: P < 0.05 between Control and Hypoxia; c: P < 0.05 between Ischemia and Hypoxia; *ND* Not detected.

**Figure 3:**
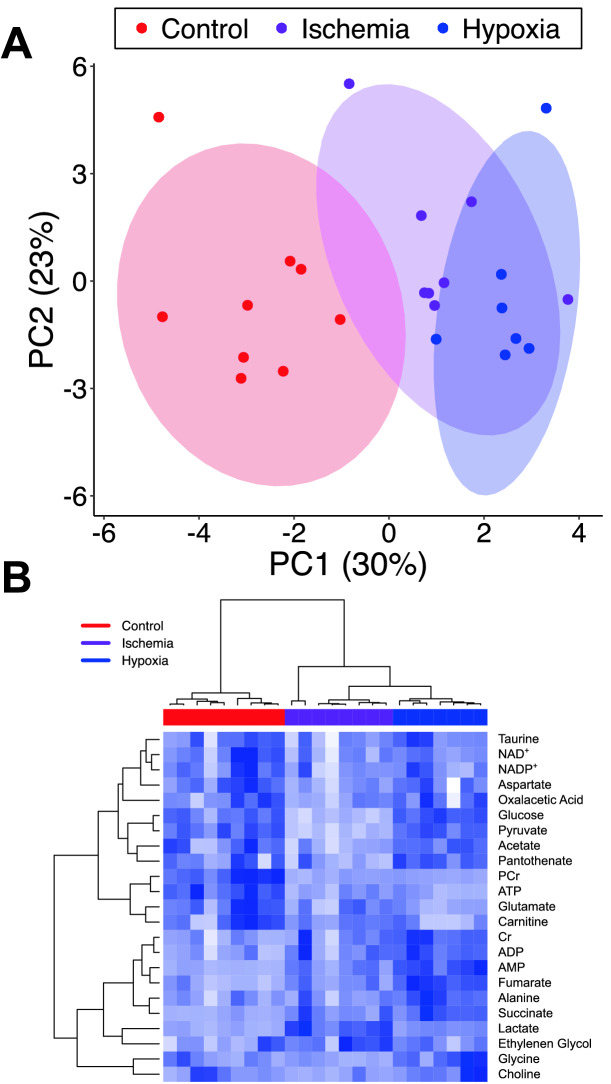
Unbiased analysis of metabolomic profiles clearly segregates ischemic hearts from controls. (**A**) Principle component analysis of the metabolomic profiles derived from ^1^H-NMR spectra clearly distinguish ischemic hearts from control hearts. (**B**) Unsupervised clustering of metabolomic data groups both hearts according to their treatment and metabolites according to their changes during ischemia.

Unbiased principle component analysis separates ischemic, hypoxic, and control hearts (Fig. 3A). Unsupervised clustering analysis (Fig. 3B) segregates hearts base on treatment and identifies groups of metabolites that track together. For example, this analysis links metabolites such as ATP and phosphocreatine, which are indicators of energetic status and tightly clusters creatine, ADP, AMP, lactate, and succinate all of which are elevated during times of declining energetic reserves. Overall these analyses confirm that the metabolomic data obtained using ^1^H-NMR spectroscopy provides metabolic signatures of ischemia or hypoxia that can be detected using non-biased methodologies.

The experimental interventions used in the current studies are expected to result in declines in energy production, and an important goal of these studies is to evaluate the relationship between declines in energy production with declines in contractile function, The energy released by ATP hydrolysis (∆G_ATP_) provides the most direct assessment of myocardial energetics. The determination of ∆G_ATP_ is complicated by the need to know the concentration of free ADP. Most ADP is bound to proteins, such as actin, and is not available to participate in chemical reactions. The free concentration of ADP can be derived from the equilibrium constant of creatine kinase. However, the creatine kinase reaction is highly dependent upon pH. Intracelluar pH is dynamic under the protocols used in the current studies ranging from 7.1 in control hearts to 6.8 in hypoxic and ischemic hearts^21,30,32,33^. The final determination of ∆G_ATP_ also requires the concentration of PO_4_. Previous studies have determined [PO_4_] to be 5 mM in control hearts, 7.5 mM in hypoxic, and 17.5 mM in ischemic hearts^21,28^.

Coupling of metabolomic data with contractile data, allows the assessment of the interaction between these data in the intact heart. Baseline contractile function was closely matched between the treatment groups (Table 2). The induction of 1 min of no-flow ischemia or 10 min of hypoxia has dramatic effects on contractile function (Table 3). The rapid decline in cardiac function following the cessation of oxygen delivery underscores the necessity of the heart to have continuous access to oxygen for energy production.

**Table 2:** Summary of baseline hemodynamic data for hearts in both control and ischemic treatment groups.

	Control (9)	Ischemia (8)	Hypoxia (7)
Diastolic pressure (mmHg)	11.9 ± 2.4	11.5 ± 2.1	14.6 ± 2.5
Systolic pressure (mmHg)	109.0 ± 7.8	113.6 ± 5.8	116.7 ± 7.3
Developed pressure (mmHg)	97.1 ± 8.0	102.1 ± 7.1	102.0 ± 9.1
Maximum dP/dt (mmHg/s)	4303.4 ± 195.8	4073.4 ± 410.6	4920.9 ± 429.2
Minimum dP/dt (mmHg/s)	− 3006.3 ± 132.8	− 2980.9 ± 352.9	− 3670.4 ± 309.8
RR interval (ms)	132.5 ± 10.3	177.6 ± 27.6	141.4 ± 2.1
Rate pressure product (mmHg/min)	43,649 ± 2480	38,817 ± 4501	43,407 ± 3801
Coronary flow (ml/min g)	127.8 ± 9.1	97.7 ± 11.0	118.8 ± 17.1
Oxygen consumption (µmol O_2_/min g)	52.9 ± 5.4	40.3 ± 4.7	64.6 ± 7.2
Cardiac efficiency (mmHg/µmol O_2_)	903.5 ± 106.1	978.7 ± 71.7	685.6 ± 44.9

Data mean ± SEM, number of observations in table header.

**Table 3:** Summary of contractile data following 1 min of ischemia or maintained perfusion with or without hypoxia.

	Control (9)	Ischemia (8)		Hypoxia (7)		Stat
Diastolic pressure (mmHg)	12 ± 2.2	3.9 ± 1.2		24 ± 4.0		b,c
Systolic pressure (mmHg)	107 ± 7.0	17.0 ± 2.8		74 ± 6.0		a,b,c
Developed pressure (mmHg)	95 ± 7.5	13.1 ± 2.1		50 ± 7.4		a,b,c
Maximum dP/dt (mmHg/s)	4233 ± 213.8	1117.2 ± 181.3		3813 ± 783.7		a,c
Minimum dP/dt (mmHg/s)	− 2927 ± 120.3	− 856.0 ± 160.7		− 1822 ± 148.6		a,b,c
RR interval (msec)	135 ± 11.4	383.8 ± 127.6		225 ± 54.8		
Rate pressure product (mmHg/min)	42,524 ± 2941	3060 ± 524		15,041 ± 1696		a,b,c
Coronary flow (ml/min g)	124 ± 9.1	NA		154 ± 11.7		b
Oxygen consumption (µmol O_2_/min g)	52 ± 5.6	NA		39 ± 3.0		
Cardiac efficiency (mmHg/µmol O_2_)	906 ± 115.0	NA		390 ± 33.5		b

Data mean ± SEM, derived from 7 and 8 hearts for Control and Ischemia conditions respectively. a: P < 0.05 between Control and Ischemia; b: P < 0.05 between Control and Hypoxia; c: P < 0.05 between Ischemia and Hypoxia.

**Figure 4:**
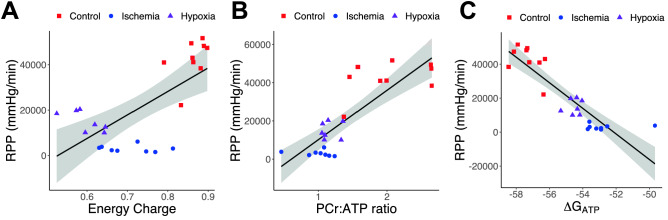
Correlations between energetic state and contractile function. Shown are the correlations rate pressure product (RPP and energetic status measured by phosphocreatine(PCr):ATP ratio (**A**–**C**), adenosine nucleotide energy charge (**D**–**F**), and ∆G_ATP_ (**G**–**I**). PCr:ATP and ∆G_ATP_ provide the best correlation to contractile function.

A means of estimating the cellular energetics is the adenosine nucleotide energy charge^24^. This parameter is poorly correlated with measures of contractile function (Fig. 4A), especially under ischemic conditions. The creatine kinase system provides a means of rapidly replenishing ATP levels. If rates of ATP synthesis remain below the levels of ATP consumption, the levels of phosphocreatine may begin to decline, even if ATP levels don’t change. Thus, the ratio of phosphocreatine to ATP is used as a measure of myocardial energetics in many studies. Not unexpectedly, the PCr:ATP ratio drops dramatically in ischemic and hypoxic hearts, to 0.99 ± 0.09 and 1.16 ± 0.05, respectively, from 2.03 ± 0.17 in control hearts. Importantly, the decline in PCr:ATP ratio is also correlated with declines in contractile function (Fig. 4B). A final form of analysis is to calculate the free energy of ATP hydrolysis (∆G_ATP_), which sets an upper limit on the energy available for ion transport and contractile function. This measure is highly correlated with contractile data providing large separations between control and ischemic hearts (Fig. 4C). Overall ∆G_ATP_ and PCr:ATP ratio provided estimates of myocardial energetics that best correlated with cardiac contractile function with r^2^ values of 0.78 and 0.68, respectively.

## Discussion

One of the central objectives of this work was to develop a means to quickly assess the energetic status of contracting myocardium. There is a long history of using ^31^P-NMR spectroscopy to measure ATP, phosphocreatine, and other biological compounds containing phosphate^4–8,34^. These methodologies are powerful in that they provide measures of these compounds in intact tissues. However, they do not provide any information on the levels of metabolites that do not contain phosphate. The field of cardiac metabolomics has blossomed in the past several years with a focus on the identification of a broader cross-section of metabolites. Many of these metabolomic studies have relied heavily on mass spectrometry to identify compounds of interest^35–40^. This approach provides a very sensitive means to identify a large number of metabolites and allows their comparison between samples. Mass spectrometry has limitations in quantifying the relative levels between metabolites within a sample. However, it is these types of comparisons that are necessary to establish a broad energetic profile of the myocardium. To address these technical limitations, many studies utilize both mass spectrometry and ^1^H-NMR spectroscopy^41–45^. This approach allows the strengths of both methodologies to be exploited to build a detailed metabolomic characterization of a tissue. However, these studies have been unable to adequately quantify the adenosine nucleotide concentrations.

The inability to distinguish between ADP and ATP by ^1^H-NMR results from the frequent overlap of their respective signals^45–47^. Currently, to determine the relative concentrations of adenosine nucleotides many studies utilize HPLC coupled to a UV-detector that allows the compounds to be identified by retention time and quantified based on standard curves^45,47^. In the current manuscript we describe relatively simple alterations in the buffering conditions used to measure the ^1^H-NMR spectra to provide sufficient peak separation to allow the identification and quantification of the adenosine nucleotides in a single assay. The combination of a robust buffering capacity coupled with the chelation of divalent cations, primarily Mg^2+^, stabilizes the chemical shifts allowing the peaks to be measured independently. In our workflow, we perform spectral scans initially in the absence of EDTA, using these spectra for the identification and quantification of nearly all of the metabolites. The peaks for ADP and ATP are often overlapping in these spectra. Adequate peak separation is achieved by the addition of EDTA, which has no ^1^H-NMR signal near the peaks of ADP and ATP.

Another critical aspect of all metabolomic studies is the details of tissue handling. This is especially true in the heart, where significant shifts in metabolite levels occur on the order of seconds. The methods of freezing tissue have been extensively studied and it is clear that speed is of the essence. Many studies use custom made tongs that have large metal blocks that can be cooled with liquid nitrogen and facilitate rapid heat transfer resulting in rapid cooling of the tissue. In preliminary studies, we found this method to be unsatisfactory with small tissues such as mouse hearts, resulting in delays in tissue freezing, raising concerns about the validity of the subsequent metabolite assessment. In these studies, we have made use of liquid nitrogen cooled 2-methyl butane to result in rapid freezing of the mouse myocardium. Evidence of the effectiveness of this approach are best demonstrated by the preservation of the PCr:ATP ratio in the perfused heart samples, which is equivalent to that determined using ^31^P-NMR approaches^28–31^.

The methodology described in this manuscript provides an important advancement allowing the assessment of myocardial energetic and metabolic status in a single assay. These measures of myocardial energetics are correlated to functional data obtained from the heart just prior to freezing. In these studies, we have demonstrated that ∆G_ATP_ and the PCr:ATP ratio have significant correlation with measures of contractile function, with the adenosine nucleotide energy charge providing a less predictive measure of heart function. While the ∆G_ATP_ provides the measure of myocardial energetics that most closely correlates with the contractile function of the myocardium, the requirement to know both pH and phosphate levels presents additional challenges in preparations or models where these parameters have not been determined. The calculations presented here rely on previous studies of wild type hearts to provide myocardial pH and phosphate levels at baseline and after a short period of ischemia or hypoxia. While this is sufficient for the studies presented here, it presents difficulties for the assessment of the energetic status of other genetic models of heart disease or different physiological challenges. Alternatively, the PCr:ATP ratio provides similar correlations to cardiac function but can be determined entirely from the methods proposed here, providing a valuable means of assessing myocardial energetics.

This method uses whole tissue homogenization, which means that only a snapshot of myocardial metabolites is acquired. Furthermore, the metabolites observed are derived from all of the cells within the organ. It is well recognized that there is a high degree of cellular heterogeneity within the heart. However, it is important to note that cardiac myocytes make up the vast majority of the actual cytoplasmic volume. As such, the metabolites extracted from the heart come primarily from cardiac myocytes. The use of perfused hearts also limits any contribution of metabolites from the extracellular components to a couple of defined metabolites; glucose and pyruvate in these studies. The perfusion of the hearts allows the reestablishment of energetic homeostasis and contractile function after the tissue has been harvested. Furthermore, this model provides a unique means of comparing cardiac energetics with myocardial contractile function.

In addition to the methodological advances, we also identify several differences in metabolite levels between ischemic and hypoxic hearts. The elevations in myocardial lactate in ischemic hearts relative to lactate is an expected consequence of the lack of perfusion of the former. However, the significant increase in a variety of anapleurotic substrates in the hypoxic heart point to additional differences in the metabolite profile of these two models. The relative increase in tricarboxylic acid (TCA) cycle intermediates (succinate, fumarate, and oxaloacetate) suggest a backup within the TCA cycle at the level of addition of new acetate groups, consistent with inhibition of pyruvate dehydrogenase. These changes likely result from the low level of oxygen delivery in hypoxic hearts allowing a small flux through the TCA cycle. While in ischemic hearts these metabolites may be siphoned off to support anaerobic metabolism. The mechanism of the significant increase in alanine and glycine in the hypoxic heart is less clear.

Another important aspect of this work is that it will allow many more laboratories to perform experiments assessing myocardial energetics. ^31^P-NMR methods require that all measurements of physiological function be made inside the NMR spectrometer, greatly limiting the nature of the functions that can be assessed. The techniques described here allow for a wide range of detailed physiological functions to be characterized, followed by snap-freezing of the tissue which allows for the subsequent analysis of the tissue’s metabolic profile. Furthermore, this approach uses standard ^1^H-NMR spectrometers that are present in a large number of core facilities. Whereas ^31^P-NMR experiments require expensive special purpose wide bore probes and a perfusion system that pushes salt solutions into the center of the spectrometer, both of which limit the feasibility of utilizing shared core facilities. These methods do present some limitations, primarily is that to collect metabolomic data the sample must be destroyed thus eliminating the ability to perform pre- and post-treatment assessments in the same heart. Another limitation, relative to ^31^P-NMR, is that because our methods provide only steady state measurements it is not possible to perform magnetization transfer experiments that allow the calculations of energetic fluxes in intact hearts.

In summary, we present a new methodology that allows the reliable identification of nearly two dozen highly abundant compounds from mouse hearts using ^1^H-NMR spectroscopy. We demonstrate that amongst these compounds are all of the adenosine nucleotides, phosphocreatine, and creatine; which together provide a detailed assessment of the energetic profile that is significantly altered by 1 min of no flow ischemia. These methods allow the correlation of metabolite levels with contractile function.
